# Association between childhood invalidation and borderline personality symptoms: self-construal and conformity as moderating factors

**DOI:** 10.1186/s40479-018-0096-6

**Published:** 2018-12-07

**Authors:** Shian-Ling Keng, Chang Yuan Soh

**Affiliations:** 1 Division of Social Science, Yale-NUS College, Singapore, Singapore; 2 Department of Psychological Medicine, National University of Singapore, Singapore, Singapore

**Keywords:** Borderline personality disorder, Childhood invalidation, Parenting, Culture, Conformity, Self-construal

## Abstract

**Background:**

Linehan (1993)‘s biosocial model posits that borderline personality disorder (BPD) symptoms develop as a result of a transactional relationship between pre-existing emotional vulnerability and an invalidating childhood environment. Little work, however, has investigated cultural factors that may influence the relationship between childhood invalidation and BPD symptoms. The present study investigated the association between parental invalidation and BPD symptoms, and the role of conformity and self-construal as potential moderators of this association.

**Methods:**

Two hundred and ninety undergraduate students were recruited from a large university in Singapore and administered questionnaires measuring Asian values, self-construal, parental invalidation, and BPD symptomatology.

**Results:**

Multiple regression analysis demonstrated a significant positive association between BPD symptoms and maternal invalidation. Moderation analyses revealed a 3-way interaction, indicating that the maternal invalidation and BPD symptoms association varied by degree of conformity and self-construal. Among participants with interdependent self-construal, maternal invalidation was associated with BPD symptoms only at high conformity levels. No significant moderating effect was found among participants with independent self-construal.

**Conclusions:**

Overall, the study found empirical support for aspects of Linehan’s biosocial model in an Asian context, and has implications for developing a culturally-informed understanding of BPD.

**Electronic supplementary material:**

The online version of this article (10.1186/s40479-018-0096-6) contains supplementary material, which is available to authorized users.

## Background

Borderline personality disorder (BPD) is a psychological disorder characterized by pervasive patterns of dysregulation in affective, interpersonal, behavioral, and cognitive domains. BPD affects approximately 0.5% [1] to 5.9% [2] of the general population, and is known as a disorder associated with elevated suicidal risks and significant psychosocial impairment [3, 4].

One influential model of BPD’s etiology is Linehan’s biosocial model [5]. The biosocial model posits that symptoms of BPD are a reflection of difficulties with emotion regulation (or emotion dysregulation). This emotional dysregulation evolves out of an on-going, transactional relationship between pre-existing emotional vulnerability and an invalidating childhood environment. Emotional vulnerability refers to the individual’s biological predisposition for unstable and intense negative affect, with high sensitivity to emotions and a slow return to baseline emotionality. An invalidating environment, on the other hand, refers to an environment that persistently disregards, ignores, or punishes an individual for expressing his or her needs and emotions. Examples of forms of invalidation include physical, sexual and emotional abuse, pervasive criticizing, minimizing, trivializing and punishing of the individual, and routine pathologizing of the individual as possessing socially undesirable personality traits [6, 7]. Invalidation may also occur in ways that are more subtle, for example, via intolerance of expression of emotional experience or oversimplifying problem solving when the child is not capable of accomplishing a particular task. Experiences of invalidation may result in individuals internalizing such behaviors (e.g., engaging in self-invalidation) and failing to learn adaptive ways of regulating emotions. As a result, individuals may resort to maladaptive ways of coping with negative emotions, such as recurrent self-injurious behaviors, which is a common feature of BPD.

To date, the role of invalidation in the etiology of BPD has received some empirical support. The majority of these studies were conducted in Western contexts such as Europe, North America, or Australia. In a sample of 202 college students based in the U.S., Cheavens and colleagues found that perceived parental criticism (a form of invalidation) was positively associated with BPD features, and this relationship was partially mediated by thought suppression (a maladaptive emotion regulation strategy) [8]. Another cross-sectional study by Sturrock and colleagues found support for a multiple mediational model, in which poor distress tolerance (a facet of emotional vulnerability) and emotional regulation difficulties mediated the association between invalidation and BPD symptoms [9].

Further, there is emerging work suggesting that the effects of invalidation on BPD symptoms may vary by parent’s gender. Previous research has highlighted the relative importance of the mother (versus the father) as the primary caregiver [10] in contributing to an environment of invalidation and the subsequent development of BPD [11, 12]. For example, a study by Sturrock and colleagues found that maternal invalidation, as opposed to paternal invalidation, significantly predicted BPD symptoms in a nonclinical sample [12]. In another study, it was found that BPD symptoms were associated with maternal overprotection (e.g., invasion of privacy), but not paternal overprotection [11]. Meanwhile, in a large sample of female undergraduates, Reeves and colleagues found no association between parental invalidation and BPD symptoms [13]. It remains to be examined whether the association between parental invalidation and BPD symptoms would emerge more consistently, should the constructs of maternal versus paternal invalidation be assessed separately.

While selected findings allude to the idea that maternal invalidation may result in more adverse consequences compared to paternal invalidation, existing literature on parent-child relationships highlight an increasingly complex ecological context where both mothers and fathers exert influence over children’s development [14–16]. For example, it has been argued that father’s parenting behaviors affect children’s outcomes in ways that are similar to mother’s parenting behaviors [17]. In a study by McDowell and Parke, both mothers’ and fathers’ parental behaviors (e.g., advice-giving and support) predicted children’s social competence and social acceptance from peers one year later [18]. It remains to be investigated whether the parent’s gender may differentially influence specific domains of children’s development, such as emotion regulation, which is known as a core deficit in the presentation of BPD [4].

### Invalidation and BPD in the Asian context

As highlighted above, empirical support for the biosocial model derives mainly from studies conducted in Western samples. Research has shown that the presentation and etiology of psychopathology varies by culture [19]. Little work to date has examined the etiology and correlates of BPD in the Asian context. Huang and colleagues recruited a sample of 400 Chinese adults and found that compared to individuals with other personality disorders and those without personality disorders, those who received a BPD diagnosis reported higher levels of parental physical, emotional, and sexual abuse [20]. In a separate study involving Chinese outpatients, Zhang and colleagues found that BPD symptomatology correlated positively with childhood emotional, physical and sexual abuse, as well as childhood emotional and physical neglect [21].

While these findings provide some support for the biosocial model, none of the studies specifically assessed the broader construct of childhood invalidation in relation to BPD symptomatology. Further, given the heterogeneity of cultures within Asia, these findings may not be generalizable to the Southeast Asian context [22]. To date, no study has yet examined the role of parental invalidation in the development of BPD symptoms in a Singaporean context – a multicultural society influenced by Confucius values, as well as other Southeast Asian heritages such as Malay and Indian cultures [23]. In Singapore, it has been found that mothers tend to be the primary caregivers, although the disciplining of children is often shared by fathers as well [24]. Further, a longitudinal study showed that maternal warmth (versus paternal warmth) uniquely predicts emotional adjustment among children in China, a country that shares cultural similarities with Singapore [25]. Taken together, the findings suggest that invalidation from the mother (versus paternal invalidation) may have a stronger impact on the development of emotional dysregulation and BPD symptoms. Therefore, we predicted that relative to paternal invalidation, maternal invalidation would be more strongly associated with BPD symptoms in Singapore.

### Exploring the role of cultural factors in the etiology of BPD

Culture can be broadly defined as a set of psychosocial processes that differentiate one group from another, which include rituals, customs, institutions, beliefs and values [26]. Given existing research that has demonstrated the complex influence of culture on cognition [27], affect [28], behavior [29], and how mental disorders develop and are expressed [19], it is plausible that specific cultural variables may be implicated in the etiology of BPD. Two cultural variables of interest are self-construal and conformity.

Several studies have investigated the role of self-construal in relation to mental health outcomes such as depression and anxiety [30, 31], but little work has explored the potential relevance of self-construal for understanding the etiology of BPD symptoms. Self-construal is a dimension of culture that pertains to the extent to which individuals understand the self as a unique, separate entity (independent self-construal), or as being defined by important, close relationships to others (interdependent self-construal) [32, 33]. Individuals with an independent self-construal tend to define themselves by their own, unique set of internal attributes (e.g., values, motives, goals), while those with an interdependent orientation tend to perceive themselves in reference to others’ feelings, thoughts, and wishes [34]. These two forms of self-construal parallel broader cultural dimensions of Individualism versus Collectivism. Whereas individualistic societies emphasize values related to autonomy, freedom, and personal traits and preferences, collectivistic societies prioritize values such as conformity to norms and emotional control in service of culturally important goals such as social harmony, smooth interpersonal functioning, and duty to one’s ingroups [35, 36].

It is notable, however, that an individual’s self-construal may not always correspond to the prevailing culture’s position on the Individualism-Collectivism dimension [37]. Endorsing a particular self-construal orientation does not automatically imply that one would subscribe to values that are typically associated with the larger cultural context. For example, it is possible that an individual living in a largely collectivist society endorses interdependent self-construal, and yet does not value conformity highly. Therefore, endorsement of self-construal and the extent to which one values conformity are related yet dissociable constructs. In this study, we define conformity as the tendency to conform to family and social norms and expectations, a trait conceptualized to be an important dimension of Asian values [38].

As highlighted above, early experience of invalidation has been theorized as an important etiological factor in BPD [5]. Beyond the objective act or behavior reflective of invalidation, the way through which an act of invalidation is *perceived* or *interpreted* may serve to exacerbate or buffer the negative impact of invalidation on emotion dysregulation and related BPD symptoms. We propose that self-construal and conformity are two factors that influence how individuals perceive, or respond to experiences of invalidation. With regards to conformity, we postulate that individuals who value conformity to norms may be more adversely affected by experiences of childhood invalidation, compared to those who do not value conformity as highly. Such individuals may experience the interpersonal obligations to conform more strongly, and perceive experiences of invalidation as a personal failure to fulfill those obligations. As a result, they may be more vulnerable to experiencing dysregulation and related BPD symptoms resulting from early experiences of invalidation. This is particularly so as parents, or primary caregivers, are the main agents of norm socialization in childhood [39]. Conversely, individuals who do not value conformity highly may not be as adversely affected by experiences of invalidation, as the perceived obligation to conform or adhere to others’ expectations may not be as strong. In the face of similar experience or history of invalidation, individuals who endorse high levels of conformity may therefore demonstrate greater symptoms of BPD, compared to those who endorse low levels of conformity. In fact, existing literature shows that a high level of family conformity orientation (the extent to which parents impose the value of conformity in children) is positively related to depression and conversely related to self-esteem in young adults and children respectively, suggesting that conformity may be a risk factor in the development of mental health issues [39, 40]. No research however has yet examined the role of conformity (as endorsed by the individual) in relation to experiences of invalidation and BPD symptoms.

As self-construal exerts a pervasive influence on how an individual relates to the self and the world [34], the effect of conformity on the association between invalidation and BPD symptoms may vary as a function of an individual’s self-construal. An individual who possesses a largely independent self-construal is less likely to value interpersonal obligations towards others strongly [35], which, in a way, may protect them from the negative impact of invalidation. For these individuals, the extent to which conformity is valued may have less of a bearing on how experiences of invalidation impact them. However, arguably, adopting a predominantly independent self-construal in a largely collectivist society (e.g. Singapore) [41] may render an individual vulnerable to experiences of invalidation, due to the inconsistency between his/her own values and the society’s values, as suggested by prior research demonstrating that a lack of fit between one’s personality and a society’s values predisposes one to having poor mental health [42]. Therefore, it is an exploratory question how experiences of invalidation would be associated with BPD symptoms for individuals with an independent self-construal.

In contrast, the association between invalidation and BPD symptoms for individuals with an interdependent self-construal may vary more as a function of conformity. An individual characterized by interdependent self-construal is likely to deem interpersonal obligations as important [35], which may predispose them to being vulnerable to experiences of invalidation and related symptoms of BPD, especially if they also value conformity to norms highly. Meanwhile, those who do not value conformity as highly may be less negatively impacted by experiences of invalidation. For these individuals, adopting an interdependent self-construal may even serve as a protective mental health factor in a society that values collectivism [42]. Therefore, it is plausible that conformity and self-construal may exert an interactive effect on the relationship between childhood invalidation and BPD symptoms. A comprehensive examination of the association among conformity, invalidation, and symptoms of BPD needs to take into account self-construal as a factor that may enhance or diminish the impact of conformity on the relationship between invalidation and BPD symptoms.

Within the larger literature, research has demonstrated a positive association between an interdependent self-construal and psychological symptoms, suggesting that individuals who are more focused on meeting duties, obligations, and social responsibilities associated with the group to which they belong (versus asserting their autonomy, needs, and wishes) may be more at risk for developing mental distress generally [43, 44]. We postulate that one way through which interdependent self-construal affects the development of mental distress may be via its influence on individuals’ responses to experiences of invalidation from the group (e.g., parents). To date, no research has yet examined type of self-construal as a potential moderator of the association between perceived invalidation and BPD symptoms.

### Specific aims and hypotheses

The present study aimed to investigate the association between parental invalidation and BPD symptoms in the Singaporean context, as well as explore the role of self-construal and conformity as potential moderators of the association. Following Reeves and colleagues [13], we adopted a dimensional perspective of BPD symptoms and recruited a nonclinical sample of college students, as late adolescence and young adulthood represents a developmental period whereby the symptoms of BPD tend to peak [45]. Based on previous research, it was hypothesized that maternal invalidation would be more strongly and positively correlated with BPD symptoms, as compared to paternal invalidation. We further hypothesized that the association between invalidation and BPD symptoms would be moderated by the interaction between self-reported degree of conformity to norms and self-construal. Specifically, we predicted that the association between invalidation and BPD symptoms would be stronger at high levels of conformity (versus low levels of conformity) among individuals with an interdependent self-construal. It was an exploratory question the extent to which invalidation would be associated with BPD symptoms, and whether the association would be moderated by conformity, among individuals with an independent self-construal.

## Methods

### Participants

Two hundred and twenty-nine undergraduate students (72% female) from National University of Singapore (NUS) participated in this study. There were no exclusion criteria. Participants’ mean age was 19.94 years old (age range = 18–31 years). The majority of participants identified as Chinese (89.7%), followed by Indian (5.2%), Malay (3.1%), and others (2.1%). Most participants identified as never married (90.3%) and were not employed (87.2%) at the time of the study.

### Procedure

Participants were recruited from Department of Psychology’s undergraduate subjects pool at NUS. Upon providing informed consent, participants completed a battery of questionnaires administered through an online platform (Qualtrics) in hour-long group sessions. The questionnaires were all administered in English. At the end of the sessions, participants were debriefed about the aims of the study and given course credits for their participation. This study was approved by NUS’ Institutional Review Board.

### Measures

#### Demographic data

The demographic information collected from participants included their gender, age, ethnicity, current relationship status, and employment status.

#### BPD symptoms

The Personality Assessment Inventory - Borderline Features Scale (PAI-BOR) is a 24-item dimensional measure designed to measure the features of severe personality pathology associated with BPD, namely, affective instability, identity problems, negative relationships, and self-harm [46]. The scale is rated on a 4-point Likert scale, ranging from 0 (“false, not true at all”) to 3 (“very true”). Examples of items include: “I’m careful about how I spend my money” and “Sometimes I feel terribly empty inside”. Higher scores indicate greater BPD symptomatology. The scale has demonstrated excellent psychometric properties, with a high internal consistency in nonclinical samples [47]. In this study, the scale demonstrated good internal consistency (Cronbach’s alpha = .86).

#### Experiences of invalidation

The Invalidating Childhood Experiences Scale (ICES) is a 28-item scale designed to measure the extent of invalidation experienced by an individual up to the age of 18 [48]. The items assess invalidating behaviors exhibited by the mother, as well as the father. Items are rated a 5-point Likert scale, ranging from 0 (“never”) to 4 (“all the time”). Examples of items include: “My [father or mother] would become angry if I disagreed with them” and “My [father or mother] would understand and help me if I couldn’t do something straight away”. Higher scores indicate greater perceived invalidation from the parent in question. In this study, the Cronbach’s alpha for the paternal scale and maternal scale was .70 and .72 respectively.

#### Self-construal

The Singelis Self-Construal Scale (SSCS) is a psychometrically validated 30-item scale developed to measure an individual’s self-construal based on Markus and Kitayama’s framework [32, 34]. Items are rated on a 7-point Likert scale, ranging from 1 (“strongly disagree”) to 7 (“strongly agree”). Examples of questions include “I enjoy being unique and different from others in many respects” and “Even when I strongly disagree with group members, I avoid an argument”. The measure consists of 2 subscales assessing independent self-construal and interdependent self-construal respectively. An overall score of independent self-construal was derived by subtracting scores from the interdependence self-construal subscale from scores from the independent self-construal subscale. A positive score reflects a primarily independent self-construal, whereas a negative overall score reflects a primarily interdependent self-construal. In this study, the scale demonstrated acceptable internal consistency (Cronbach’s alpha = .76).

#### Conformity

The Asian Values Scale-Revised (AVS-R) is a 25-item scale developed to assess Asian values, conceptualized to encompass conformity to norms (conformity), family recognition through achievement, emotional self-control, collectivism, humility and filial piety [38, 49]. Items are rated on a 7-point Likert scale, ranging from 0 (“strongly disagree”) to 6 (“strongly agree”). Example questions include: “One should not deviate from familial and social norms” and “One need not control one’s expression of emotion”. Higher scores indicate greater endorsement of Asian values. The scale has been validated in Asian populations in the USA and demonstrate high levels of internal consistency and test-retest reliability across 2 weeks [38]. We administered this measure to assess the construct of conformity, given the lack of other established measures that assess this construct at the time of the study. As there are no pre-established, built-in subscales and factor structures for AVS-R, we conducted a principal components analysis to derive items that assess conformity specifically (see below for results). In this study, the AVS-R demonstrated acceptable internal consistency (Cronbach’s alpha = .70).

### Data analytic plan

All analyses were performed using SPSS Version 22.0. Data were cleaned and checked for outliers prior to analyses. There were no missing data. We first ran a principal components factor analysis with a varimax rotation on AVS-R to derive items that assess the conformity factor. Items with a minimum factor loading of .3 and factors with a minimum of an eigenvalue of 1.00 and 3 items loaded were retained.

To examine the association between childhood invalidation (both maternal and paternal) and BPD symptoms, BPD symptoms were regressed onto paternal and maternal childhood invalidation separately, and thereafter simultaneously. To examine potential moderating effects of conformity and self-construal on the association between invalidation and BPD symptoms, a multiple regression was run using the PROCESS macro [50]. PAI-BOR scores were regressed onto either maternal or paternal invalidation (depending on the factor that emerged as significant in the prior regression model), self-construal (overall score of independent self-construal derived from subtracting scores from the interdependence self-construal subscale), conformity, the self-construal × invalidation term, the conformity × invalidation term, the self-construal × conformity term, and the 3-way self-construal × invalidation × conformity interaction term. All variables were mean centered.

## Results

### Preliminary analysis

Principal components factor analysis with an orthogonal varimax rotation applied onto the AVS-R demonstrated an 8-component solution accounting for 60.40% of the variance (see Additional file 1: Appendix A for results of the factor analysis). Following exclusion of items with a factor loading of less than 0.3, and factors with less than 3 items loaded on them, there were 19 items loaded onto five components. Content analyses of the items indicate that the components correspond to: Family’s “Face” concerns, Academic Achievement, Humility and Modesty, Authority Adherence, and Conformity to Norms respectively. Items for the Conformity to Norms factor were averaged into a subscale score, with higher scores reflecting a greater endorsement of conformity.

### Primary analyses

#### Association between childhood invalidating experiences and BPD symptoms

Results from simple regression analyses showed that both maternal invalidation (*F*(1, 288) = 30.15, *B =* 5.47*, SE =* .99, *p* < .001, f^2^ = .11) and paternal invalidation (*F*(1, 288) = 21.25, *B =* 4.65*, SE =* 1.00, *p* < .001, f^2^ = .07) individually predicted BPD symptoms. When both predictors were entered into a multiple regression model, maternal invalidation remained as a significant predictor, *B =* 4.43*, SE =* 1.45, *p* = .003, f^2^ = .10, whereas paternal invalidation did not predict BPD symptoms, *B =* 1.43*, SE =* 1.45, *p* = .33, f^2^ = .003. Together, the two predictors explained 10% of the variance associated with BPD symptoms, *F*(2, 287) = 15.56, *p* < .01. Further analyses were conducted with maternal invalidation retained in the model.

#### Self-construal and conformity as moderators

Results from moderation analyses demonstrated that PAI-BOR scores were predicted by maternal invalidation, *B =* 4.63*, SE =* 1.03, *p* < .0001, f^2^ = .10, self-construal, *B =* −.14*, SE =* .05, *p* = .005, f^2^ = .02, and the self-construal × conformity interaction, *B =* .04*, SE =* .02, *p* = .02, f^2^ = .01. The interaction was qualified by a significant 3-way self-construal × invalidation × conformity interaction, *B =* −.06*, SE =* .02, *p* = .02, f^2^ = .02. None of the other predictors in the model (conformity, the self-construal × invalidation term, the conformity × invalidation term) was statistically significant, all *p*s >. 14.

**Fig. 1:**
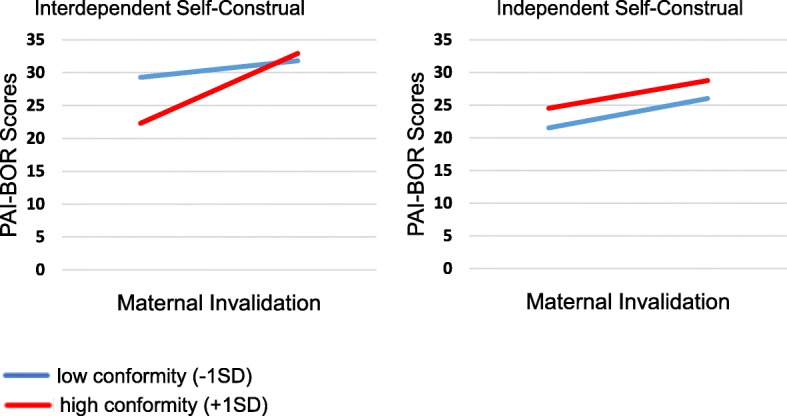
Three-way interaction among self-construal, conformity, and maternal invalidation, with the left and right panels demonstrating the conformity by maternal invalidation interaction for participants with predominantly interdependent self-construal and those with primarily independent self-construal respectively

Given the significant 3-way interaction, we tested the conditional effects of maternal invalidation at different values of each of the moderators. For individuals with predominantly interdependent self-construal,^1^ high levels of conformity (1 *SD* above the mean) predicted a significant, positive association between maternal invalidation and BPD symptoms, *B =* 8.86*, SE =* 1.81, *p* = .0002, whereas low levels of conformity (1 *SD* below the mean) did not, *B =* 1.02*, SE =* 2.16, *p* = .56 (See Fig. 1). For individuals with predominantly independent self-construal, there was a significant association between maternal invalidation and BPD symptoms across both high (*B =* 5.02*, SE =* 1.75, *p* = .005) and low levels of conformity (*B =* 3.63*, SE =* 1.69, *p* = .03).

## Discussion

The current study sought to examine the association between parental invalidation and BPD symptoms in a Singaporean context, and self-construal and conformity as potential moderators of the invalidation-BPD symptoms association. As hypothesized, the results showed a positive association between BPD symptoms and parental invalidation, with maternal invalidation playing a larger role. Further, the results demonstrated that the association between maternal invalidation and BPD symptoms varied as a function of self-construal orientation and conformity. Specifically, conformity moderated the association between invalidation and BPD symptoms for individuals with an interdependent self-construal. Among these individuals, those who endorsed high conformity showed a significant, positive association between maternal invalidation and BPD symptoms; whereas those who endorsed low conformity levels showed no relationship between maternal invalidation and BPD symptomatology. Among individuals with an independent self-construal, the relationship between maternal invalidation and BPD symptoms was not moderated by levels of conformity.

The finding that maternal invalidation is more strongly associated with BPD symptoms, compared to paternal invalidation, is consistent with previous studies that have examined the parent-specific effects of invalidation in BPD etiology [11, 12]. This finding may be attributed to the structure of traditional families, especially in Asian cultures, whereby mothers are more often the primary caregiver [10], and spend more time with their children [51]. Further, based on traditional gender role differentiations, mothers tend to be expected to assume a caring and warm role in children’s socialization, whereas fathers may assume more disciplinary duties in general [52, 53]. For example, in an observational study involving children aged between 6 and 7 years old, mothers were found to interact with children more, as well as demonstrate greater involvement in caregiving compared to fathers [53]. Meanwhile, fathers’ interactions with children occurred more frequently in the context of play. Within this gender role context, children may respond more negatively to invalidation coming from mothers, who are expected to be warmer and more nurturing compared to fathers. Our study’s finding is consistent with that of Chang and colleagues, who found that harsh maternal parenting (a form of invalidation) contributes more to emotional dysregulation in children compared with harsh paternal parenting, in a sample of over 300 Chinese children and their families [54]. Taken together, the findings highlight the role of maternal invalidation as a potential risk factor involved in the development of emotion dysregulation and BPD symptoms.

The finding that the relationship between maternal invalidation and BPD symptoms varies as a function of self-construal and conformity provides among the first evidence for the role of culturally-relevant processes in the intersection between experiences of invalidation and BPD symptoms. In particular, among individuals with an interdependent construal, conformity significantly moderated the relationship between invalidation and BPD symptoms, such that the relationship was much stronger among those who endorse high levels of conformity. The finding can be interpreted in light of the processes through which individuals with interdependent self-construal tend to evaluate themselves [55]. Self-evaluations for interdependently-oriented individuals tend to be contingent on how their actions affect relationally-important others, as the identity of individuals with an interdependent self-construal is defined primarily by in-group memberships [55]. Such a self-evaluative orientation, coupled with a high endorsement of conformity as a value, might result in these individuals especially vulnerable to experiences of invalidation. Specifically, these individuals may perceive invalidation as sign of their failure to carry out expected duties and obligations, which exacerbates the negative effects of the invalidating experience. On the other hand, if the individual does not value conformity highly, he or she may be less likely to perceive experiences of invalidation as a sign of failure or having let down others’ expectations. Further, endorsing an interdependent self-construal orientation may serve as a protective factor against emotional distress or dysregulation, as such an orientation fits with the larger (Asian) cultural context that also values maintenance of social harmony and relationally-important goals [31, 42]. Overall, these findings are suggestive of the role of low conformity as a protective factor of the impact of invalidation on BPD symptoms among individuals with interdependent self-construal. On the other hand, conformity did not moderate the relationship between maternal invalidation and BPD symptoms among individuals with independent self-construal. Among these individuals, maternal invalidation predicted higher levels of BPD symptoms regardless of levels of conformity. The finding corresponds with the larger literature that demonstrated a positive association between maternal invalidation and borderline symptoms [12].

To the authors’ knowledge, this study represents one of the first empirical attempts to examine the intersection between BPD traits and cultural factors. The sample size of the study (*N* = 290) was adequately large to allow for reliable analyses of moderation effects. Further, recruitment of a sample based in Singapore enables generalization of the findings to an Asian cultural context. Meanwhile, there are several limitations to the present study. Importantly, the study’s design is correlational, which precludes the inference of causal conclusions. Future research should employ an experimental or longitudinal design to examine the causal association between parental invalidation and BPD symptoms, as well as how invalidation may intersect with cultural factors to influence the development of BPD symptoms. Second, the study utilized self-report measures to assess key variables of interest. Therefore, the findings could be attributable to shared method variance or other forms of self-report biases. Further, the measure of childhood invalidation was retrospective in nature, and may not accurately assess childhood experiences, due to memory biases [56]. Future studies should employ multiple methods with adequate reliability (e.g. behavioral observation) to assess parental invalidation and other related constructs. Also, the internal consistencies of several scales used in this study are not strong; future studies should validate and explore possible adaptation of the scales in the Singaporean context. Lastly, the study was conducted using a sample of undergraduate students, as opposed to a clinical sample. The findings therefore may not be generalizable to individuals with diagnosed BPD, or those with more severe psychological symptoms or BPD traits. Future research should replicate the findings in a clinical sample, as well as examine additional components of the biosocial model (e.g., pre-existing emotional vulnerability) to understand how these factors may interact with invalidating experiences and/or cultural factors in giving rise to symptoms of BPD over time.

## Conclusions

In sum, the present study provided support for aspects of the biosocial model in an Asian context. In particular, maternal invalidation, as opposed to paternal invalidation, was found to be significantly associated with BPD symptoms. Further, the study found preliminary support for the role of cultural factors, particularly self-construal and conformity, as moderators of the association between invalidation and BPD symptoms. The findings speak to the importance of taking cultural variables into consideration in the conceptualization of etiological models for BPD, as well as in case formulation when working with individuals with BPD symptoms. Future research should replicate these findings in clinical samples, examine the causal pathways underlying the association among invalidation, cultural factors, and BPD symptoms, as well as investigate the psychological mechanisms through which cultural variables play a role in the presentation and etiology of BPD symptoms.

## Additional file

Additional file 1: Appendix A. AVS-R Standardized Factor Loadings of Exploratory Factor Analysis. (DOCX 15 kb)

## Footnotes

1 It is recognized that few people are exclusively independent or interdependent in their self-construal orientation (Triandis, 1989). Therefore, we used the terms “predominantly independent” or “predominantly interdependent” to describe our sample. For brevity’s sake, we subsequently dropped the word “predominantly” when describing the self-construal orientation of our participants.

## References

[1] Samuels J, Eaton WW, Bienvenu OJ, Brown CH, Costa PT, Nestadt G (2002). Prevalence and correlates of personality disorders in a community sample. Br J Psychiatry.

[2] Grant BF, Chou SP, Goldstein RB, Huang B, Stinson FS, Saha TD (2008). Prevalence, correlates, disability, and comorbidity of DSM-IV borderline personality disorder. J Clin Psychiatry.

[3] Black DW, Blum N, Pfohl B, Hale N (2004). Suicidal behavior in borderline personality disorder: prevalence, risk factors, prediction, and prevention. J Personal Disord.

[4] Lieb K, Zanarini MC, Schmahl C, Linehan MM, Bohus M (2004). Borderline personality disorder. Lancet.

[5] Linehan MM (1993). Cognitive-behavioral treatment of borderline personality disorder.

[6] Crowell SE, Beauchaine TP, Linehan MM (2009). A biosocial developmental model of borderline personality: elaborating and extending Linehan’s theory. Psychol Bull.

[7] Wagner AW, Linehan MM, Zanarini MC (1997). Biosocial perspective on the relationship of childhood sexual abuse, suicidal behavior, and borderline personality disorder. Progress in psychiatry, no. 49. Role of sexual abuse in the etiology of borderline personality disorder.

[8] Cheavens JS, Rosenthal MZ, Daughters SB, Nowak J, Kosson D, Lynch TR (2005). An analogue investigation of the relationships among perceived parental criticism, negative affect, and borderline personality disorder features: the role of thought suppression. Behav Res Ther.

[9] Sturrock B, Mellor D (2014). Perceived emotional invalidation and borderline personality disorder features: a test of theory. Personal Ment Health.

[10] Cinamon RG, Rich Y (2002). Gender differences in the importance of work and family roles: implications for work–family conflict. Sex Roles.

[11] Arens EA, Grabe HJ, Spitzer C, Barnow S (2011). Testing the biosocial model of borderline personality disorder: results of a prospective 5-year longitudinal study. Personal Ment Health.

[12] Sturrock BA, Francis A, Carr S (2009). Avoidance of affect mediates the effect of invalidating childhood environments on borderline personality symptomatology in a non-clinical sample. Clin Psychol.

[13] Reeves M, James LM, Pizzarello SM, Taylor JE (2010). Support for Linehan’s biosocial theory from a nonclinical sample. J Personal Disord.

[14] Cabrera NJ, Volling BL, Barr R (2018). Fathers are parents, too! Widening the lens on parenting for children's development. Child Dev Perspect.

[15] Kalil A, Ryan R, Chor E (2014). Time investments in children across family structures. Ann Am Acad Pol Soc Sci.

[16] Maccoby EE (1992). The role of parents in the socialization of children: an historical overview. Dev Psychol.

[17] Fagan J, Day R, Lamb ME, Cabrera NJ (2014). Should researchers conceptualize differently the dimensions of parenting for fathers and mothers?. J Fam Theory Rev.

[18] McDowell DJ, Parke RD (2009). Parental correlates of children's peer relations: an empirical test of a tripartite model. Dev Psychol.

[19] Hwang WC, Myers HF, Abe-Kim J, Ting JY (2008). A conceptual paradigm for understanding culture's impact on mental health: the cultural influences on mental health (CIMH) model. Clin Psychol Rev.

[20] Huang J, Yang Y, Wu J, Napolitano LA, Xi Y, Cui Y (2012). Childhood abuse in Chinese patients with borderline personality disorder. J Personal Disord.

[21] Zhang T, Chow A, Wang L, Dai Y, Xiao Z (2012). Role of childhood traumatic experience in personality disorders in China. Compr Psychiatry.

[22] Salant T, Lauderdale DS (2003). Measuring culture: a critical review of acculturation and health in Asian immigrant populations. Soc Sci Med.

[23] Huat CB (2003). Multiculturalism in Singapore: an instrument of social control. Race & Class.

[24] Ebbeck M, Gokhale N (2004). Child-rearing practices in a selected sample of parents with children in childcare in Singapore. Contemp Issues Early Child.

[25] Chen X, Liu M, Li D (2000). Parental warmth, control, and indulgence and their relations to adjustment in Chinese children: a longitudinal study. J Fam Psychol.

[26] Cooper S, Leong FT (2008). Introduction to the special issue on culture, race, and ethnicity in organizational consulting psychology. Consult Psychol J Pract Res.

[27] Matsumoto D (1999). Culture and self: an empirical assessment of Markus and Kitayama's theory of independent and interdependent self-construals. Asian J Soc Psychol.

[28] Butler EA, Lee TL, Gross JJ (2007). Emotion regulation and culture: are the social consequences of emotion suppression culture-specific?. Emotion.

[29] Hoshino-Browne E, Zanna AS, Spencer SJ, Zanna MP, Kitayama S, Lackenbauer S (2005). On the cultural guises of cognitive dissonance: the case of easterners and westerners. J Pers Soc Psychol.

[30] Lam BT (2005). Self-construal and depression among Vietnamese-American adolescents. Int J Intercult Relat.

[31] Mak WW, Law RW, Teng Y (2011). Cultural model of vulnerability to distress: the role of self-construal and sociotropy on anxiety and depression among Asian Americans and European Americans. J Cross-Cult Psychol.

[32] Singelis TM (1994). The measurement of independent and interdependent self-construals. Personal Soc Psychol Bull.

[33] Triandis HC (1989). The self and social behavior in differing cultural contexts. Psychol Rev.

[34] Markus HR, Kitayama S (1991). Culture and the self: implications for cognition, emotion, and motivation. Psychol Rev.

[35] Brewer MB, Chen YR (2007). Where (who) are collectives in collectivism? Toward conceptual clarification of individualism and collectivism. Psychol Rev.

[36] Kim H, Markus HR (1999). Deviance or uniqueness, harmony or conformity? A cultural analysis. J Pers Soc Psychol.

[37] Triandis HC, Bontempo R, Villareal MJ, Asai M, Lucca N (1988). Individualism and collectivism: cross-cultural perspectives on self-ingroup relationships. J Pers Soc Psychol.

[38] Kim BS, Atkinson DR, Yang PH (1999). The Asian values scale: development, factor analysis, validation, and reliability. J Couns Psychol.

[39] Schrodt Paul, Ledbetter Andrew M., Ohrt Jennifer K. (2007). Parental Confirmation and Affection as Mediators of Family Communication Patterns and Children's Mental Well-Being. Journal of Family Communication.

[40] Hamon JD, Schrodt P (2012). Do parenting styles moderate the association between family conformity orientation and young adults’ mental well-being?. J Fam Commun.

[41] Gelfand MJ (2012). Culture’s constraints: international differences in the strength of social norms. Curr Dir Psychol Sci.

[42] Caldwell-Harris CL, Ayçiçegi A (2006). When personality and culture clash: the psychological distress of allocentrics in an individualist culture and idiocentrics in a collectivist culture. Transcult Psychiatry.

[43] Christopher MS, Skillman GD (2009). Exploring the link between self-construal and distress among African American and Asian American college students. J Coll Couns.

[44] Okazaki S (1997). Sources of ethnic differences between Asian American and white American college students on measures of depression and social anxiety. J Abnorm Psychol.

[45] Stepp SD (2012). Development of borderline personality disorder in adolescence and young adulthood: introduction to the special section. J Abnorm Child Psychol.

[46] Morey LC (1999). Personality assessment inventory: professional manual.

[47] Trull TJ (1995). Borderline personality disorder features in nonclinical young adults: 1. Identification and validation. Psychol Assess.

[48] Mountford V, Corstorphine E, Tomlinson S, Waller G (2007). Development of a measure to assess invalidating childhood environments in the eating disorders. Eat Behav.

[49] Kim BS, Hong S (2004). A psychometric revision of the Asian values scale using the Rasch model. Meas Eval Couns Dev.

[50] Hayes A (2013). Introduction to mediation, moderation, and conditional process analysis.

[51] Craig L (2006). Does father care mean fathers share? A comparison of how mothers and fathers in intact families spend time with children. Gend Soc.

[52] Phares V, Compas BE (1993). Fathers and developmental psychopathology. Curr Dir Psychol Sci.

[53] Russell Graeme, Russell Alan (1987). Mother-Child and Father-Child Relationships in Middle Childhood. Child Development.

[54] Chang L, Schwartz D, Dodge KA, McBride-Chang C (2003). Harsh parenting in relation to child emotion regulation and aggression. J Fam Psychol.

[55] Heine SJ (2001). Self as cultural product: an examination of east Asian and north American selves. J Pers.

[56] Stone AA, Shiffman S (2002). Capturing momentary, self-report data: a proposal for reporting guidelines. Ann Behav Med.

