# Supplementary material for: Association between childhood invalidation and borderline personality symptoms: self-construal and conformity as moderating factors
Source: Borderline Personal Disord Emot Dysregul. 2018 Dec 7;5:19. doi: 10.1186/s40479-018-0096-6 (PMC6284292; doi:10.1186/s40479-018-0096-6)
Supplement: Supplementary file 1 — Appendix A. AVS-R Standardized Factor Loadings of Exploratory Factor Analysis. (DOCX 15 kb) [file 40479_2018_96_MOESM1_ESM.docx]

**Appendix**

| *Additional file 1: Appendix A. AVS-R Standardized Factor Loadings of Exploratory Factor Analysis* | | | | | | | |
| --- | --- | --- | --- | --- | --- | --- | --- |
|  | Factor Loadings | | | | | | |
| Item | Family Face Concerns | Academic Achievement | | | Humility and Modesty | Authority Adherence | Conformity to Norms |
| 17. The worst thing one can do is to bring disgrace to one’s family reputation. | 0.772 |  | | |  |  |  |
| 15. One should avoid bringing displeasure to one’s ancestors. | 0.727 |  | | |  |  |  |
| 14. One’s achievements should be viewed as family’s achievements. | 0.675 |  | | |  |  |  |
| 20. Family’s reputation is not the primary social concern. (rs) | 0.659 |  | | |  |  |  |
| 23. One need not follow the role expectations (gender, family hierarchy) of one’s family. (rs) | 0.494 |  | | |  |  |  |
| 22. Occupational failure does not bring shame to the family. (rs) | 0.392 |  | | |  |  |  |
| 7. One need not achieve academically in order to make one’s parents proud. (rs) |  | 0.804 | | |  |  |  |
| 10. Educational and career achievements need not be one’s top priority. (rs) |  | 0.743 | | |  |  |  |
| 3. One need not focus all energies on one’s studies. (rs) |  | 0.588 | | |  |  |  |
| 8. One need not minimize or depreciate one’s own achievements. (rs) |  | 0.55 | | |  |  |  |
| 13. Modesty is an important quality for a person. |  |  | | | 0.787 |  |  |
| 19. One should be humble and modest. |  |  | | | 0.766 |  |  |
| 2. Children should not place their parents in retirement homes. |  |  | | | 0.491 |  |  |
| 16. One should have sufficient inner resources to resolve emotional problems. |  |  | | |  | -0.72 |  |
| 12. One should be able to question a person in a position of authority. (rs) |  |  | | |  | 0.586 |  |
| 5. Younger persons should be able to confront their elders. (rs) | | |  |  |  | 0.445 |  |
| 24. One should not disrupt the status quo. | | |  |  |  |  | 0.641 |
| 6. When one receives a gift, one should reciprocate with a gift of equal or greater value. | | |  |  |  |  | 0.617 |
| 1. One should not deviate from familial and social norms. | | |  |  |  |  | 0.446 |
| Unloaded | | |  |  |  |  |  |
| 4. One should be discouraged from talking about one’s accomplishments. | | |  |  |  |  |  |
| 21. One need not be able to resolve psychological problems on one’s own. (rs) | | |  |  |  |  |  |
| 25. One need not control one’s expression of emotions. (rs) | | |  |  |  |  |  |
| 18. One need not remain reserved and tranquil. (rs) | | |  |  |  |  |  |
| 9. One should consider the needs of others before considering one’s own needs. | | |  |  |  |  |  |
| 11. One should think about one's group before oneself. | | |  |  |  |  |  |
| Note. rs = reverse-scored.  Only the greatest loading of items, for items with a factor loading equal to or more than 0.3 and factors with equal to or more than 3 items loaded only, are displayed. | | | | | | | |
|  |  |  |  |  |  |  |  |
